# Global and local perturbation of the tomato microRNA pathway by a *trans*-activated *DICER-LIKE 1* mutant

**DOI:** 10.1093/jxb/ert428

**Published:** 2013-12-27

**Authors:** Michael Kravchik, Ramanjulu Sunkar, Subha Damodharan, Ran Stav, Matat Zohar, Tal Isaacson, Tzahi Arazi

**Affiliations:** ^1^Institute of Plant Sciences, Agricultural Research Organization, Volcani Center, PO Box 6, Bet Dagan 50250, Israel; ^2^Department of Biochemistry & Molecular Biology, Oklahoma State University, Stillwater, OK 74078, USA; ^3^Unit of Deciduous Fruit Tree Sciences, Newe Ya’ar Research Center, Agricultural Research Organization, PO Box 1021, Ramat Yishay 30095, Israel

**Keywords:** Carotenoid, DCL1, leaf, miRNA, mutant, petal, tomato.

## Abstract

DICER-like 1 (DCL1) is a major player in microRNA (miRNA) biogenesis and accordingly, its few known loss-of-function mutants are either lethal or display arrested development. Consequently, generation of *dcl1* mutants by reverse genetics and functional analysis of DCL1 in late-developing organs are challenging. Here, these challenges were resolved through the unique use of *trans*-activated RNA interference. Global, as well as organ-specific tomato *DCL1* (*SlDCL1*) silencing was induced by crossing the generated responder line (*OP:SlDCL1IR*) with the appropriate driver line. Constitutive *trans*-activation knocked down *SlDCL1* levels by ~95%, resulting in severe abnormalities including post-germination growth arrest accompanied by decreased miRNA and 21-nucleotide small RNA levels, but prominently elevated levels of 22-nucleotide small RNAs. The increase in the 22-nucleotide small RNAs was correlated with specific up-regulation of *SlDCL2b* and *SlDCL2d*, which are probably involved in their biogenesis. Leaf- and flower-specific *OP:SlDCL1IR trans*-activation inhibited blade outgrowth, induced premature bud senescence and produced pale petals, respectively, emphasizing the importance of *SlDCL1*-dependent small RNAs in these processes. Together, these results establish *OP:SlDCL1IR* as an efficient tool for analysing processes regulated by *SlDCL1*-mediated gene regulation in tomato.

## Introduction

Small RNAs, ranging from 20 to 24 nucleotides (nt) in size, have many key functions in plants, particularly in genome stability, regulation of gene expression, and defence by directing sequence-specific repression of target RNAs (Chen, 2009; Axtell, 2013*a*). Plant microRNAs (miRNAs) constitute a major class of endogenous small RNAs that have emerged as important post-transcriptional regulators of plant development, stress responses, and nutrient homeostasis (Jones-Rhoades *et al.*, 2006; de Lima *et al.*, 2012; Sunkar *et al.*, 2012). In contrast to small interfering RNAs (siRNAs), which are generated via processing of long double-stranded RNA precursors with perfect complementarity, miRNAs are processed from a single-stranded RNA precursor transcript or pri-miRNA that can form imperfect fold-back structures (Reinhart *et al.*, 2002).

The diversification of small RNA pathways in plants can be attributed in part to specific DICER-like (DCL) proteins that specialize in the production of distinct small RNAs. Four types of DCLs are present in all angiosperms (Margis *et al.*, 2006). Among them, the product of *DCL1*, also termed *SHORT INTEGUMENTS1* (*SIN1*)/*SUSPENSOR1* (*SUS1*)*/CARPEL FACTORY* (*CAF*) (Robinson-Beers *et al.*, 1992; Jacobsen *et al.*, 1999; McElver *et al.*, 2001; Schauer *et al.*, 2002), catalyses the formation of predominantly 20- to 22-nt canonical miRNAs (Park *et al.*, 2002; Reinhart *et al.*, 2002; Kurihara and Watanabe, 2004). In addition, certain inverted repeat-derived siRNAs and secondary siRNAs—including *trans*-acting siRNAs (ta-siRNAs), natural antisense siRNAs, and phased siRNAs—which do not derive from fold-back structures but arise from transcripts targeted by miRNAs, also depend on DCL1 for their accumulation (Allen *et al.*, 2005; Borsani *et al.*, 2005; Henderson *et al.*, 2006; Li *et al.*, 2012*b*; Shivaprasad *et al.*, 2012). Moreover, DCL1 was found to contribute to the silencing of a subset of transposons, apparently through an effect on DNA methylation (Laubinger *et al.*, 2010). DCL2 catalyses the formation of 22-nt siRNAs and 22- to 24-nt natural antisense siRNAs (Xie *et al.*, 2004; Borsani *et al.*, 2005; Deleris *et al.*, 2006). DCL3 is responsible for the processing of 24-nt heterochromatin-associated siRNAs (hc-siRNAs) and long miRNAs (Xie *et al.*, 2004; Qi *et al.*, 2005; Wu *et al.*, 2010). DCL4 sequentially processes 21-nt secondary siRNAs including ta-siRNAs and other phased siRNAs, and is required for the production of several *Arabidopsis thaliana* miRNAs such as miR822 and miR839 (Xie *et al.*, 2004; Gasciolli *et al.*, 2005; Rajagopalan *et al.*, 2006; Howell *et al.*, 2007).

To date, *dcl1* loss-of-function mutants have been isolated from *Arabidopsis*, *Oryza sativa*, and the moss *Physcomitrella patens* (Schauer *et al.*, 2002; Liu *et al.*, 2005; Khraiwesh *et al.*, 2010). In *Arabidopsis*, null *dcl1* alleles (*sus1*) are embryo lethal (Castle *et al.*, 1993). In addition, several hypomorphic *dcl1* mutants have been described, such as *dcl1-7* (*sin 1-1*) and *dcl1-9* (*caf*), which show pleiotropic developmental phenotypes due to reduced miRNA levels (Robinson-Beers *et al.*, 1992; Jacobsen *et al.*, 1999; Park *et al.*, 2002). *Physcomitrella* harbours two DCL1-like proteins, PpDCL1a and PpDCL1b. Deletion of *PpDCL1a* but not *PpDCL1b* drastically reduced miRNA levels, indicating its functional equivalence to AtDCL1. *ΔPpDCL1*a-null mutants showed developmental defects and abnormalities in cell size and shape; their growth was severely retarded and they were developmentally arrested at the filamentous protonema stage (Khraiwesh *et al.*, 2010). In rice, a DCL1 loss-of-function mutant (*OsDCL1IR*) was generated by RNA interference (RNAi). Silenced plants had reduced levels of miRNAs, developed severe dwarfism, rolled and curly leaves, and tortuous shoots, and were arrested at the young seedling stage (Liu *et al.*, 2005). The lethality and severe growth arrest associated with *dcl1* loss-of-function mutants prevents their functional characterization at late developmental stages. It also impedes the generation of *dcl1* loss-of-function mutants by reverse genetics in plants whose transformation includes a regeneration phase in tissue culture. For example, attempts to stably silence *Nicotiana attenuata DCL1* by RNAi have failed due to lethality of the regenerated transformants (Bozorov *et al.*, 2012).

*Solanum lycopersicum* (tomato) is one of the most important crops in the fresh vegetable market and the food-processing industry, and is a good model for crop species with fleshy berry fruits (Giovannoni, 2001). Deep sequencing of small RNAs from various tomato tissues has revealed the presence of a complex population of small RNAs, including miRNAs (Moxon *et al.*, 2008; Li *et al.*, 2012*b*; Shivaprasad *et al.*, 2012; Axtell, 2013*b*). Recently, a tomato *dcl4* mutant has been described, demonstrating its function in the biogenesis of ta-siRNAs and their involvement in the regulation of lamina expansion during compound leaf development (Yifhar *et al.*, 2012). The functions of other tomato DCLs, including DCL1, remain unstudied.

In the current study, a *trans*-activated RNAi approach was employed to silence tomato DCL1 (*SlDCL1*) globally or in specific organs, and the resulting mutants were utilized to investigate SlDCL1 functions in small RNA biogenesis and development.

## Materials and methods

### Plant material and growth conditions

The tomato (*S. lycopersicum*) cv. M82 driver lines *35S:LhG4*, *FIL:LhG4* (Lifschitz *et al.*, 2006), *AP1:LhG4*, and *AP3:LhG4* (Fernandez *et al.*, 2009; Hendelman *et al.*, 2013*a*) are described elsewhere. Tomato plants were grown under greenhouse conditions with temperatures ranging between 15 °C and 30 °C in a tuff–peat mix with nutrients, using 4 litre pots. Germination and seedling growth took place in a growth chamber with a 16h light period and 8h dark period (photosynthetic photon flux density: 50–70 μmol m^–2^ s^–1^) at a constant temperature of 24 °C. For crosses, closed flowers were emasculated by removal of the petals and stamens and hand pollinated with the pollen of an appropriate homozygous driver line.

### Sequencing of full-length *SlDCL1* and *SlDCL2a*, *b*, and *d* 5′-untranslated regions (UTRs)

To determine the full-length sequence of *SlDCL1*, total RNA was extracted from tomato cv. M82 flowers at anthesis as described below and a 3′ and 5′ RNA ligase-mediated rapid amplification of cDNA ends (RLM-RACE) procedure was performed on this RNA. The partial *Solyc10g005130* sequence was used to design gene-specific primers (all primer sequences are given in Supplementary Table S1 available at *JXB* online) as follows: for the 5′ end, SlDCL1-5′-RACE and SlDCL1-5′-RACE-nested; for the 3′ end, SlDCL1-3′-RACE and SlDCL1-3′-RACE-nested. The resulting amplified products were gel-purified, cloned, and sequenced. After assembling the full-length sequence of *SlDCL1*, its 5′ (nt 86–2685) and overlapping 3′ (nt 2481–6168) cDNA sequences were PCR-amplified with SlDCL1_F_XhoI and SlDCL1_R_2685, and SlDCL1_F_2481 and SlDCL1_R_6168, respectively, and sequenced. The 5′ UTR of *SlDCL2b* (*Solyc11g008540*) was determined by RLM-RACE of total flower RNA with SlDCL2b-5′-RACE and SlDCL2b-5′-RACE-nested primers. On the basis of their sequence, the 5′ UTRs of *SlDCL2a* (*Solyc06g048960*) and *SlDCL2d* (*Solyc11g008530*) were determined by PCR on flower cDNA with the primer pairs SlDCL2a_F_28 and qRT-SlDCL2a_R, and SlDCL2d_R_280 and qRT-SlDCL2d_F, respectively. Amplified products were then gel-purified and sequenced directly.

### Plasmid construction

For the *SlDCL1IR* RNAi reporter construct, a 674bp *SlDCL1* sequence composed of 264bp and 410bp of its promoter and cDNA, respectively, was amplified by two-step PCR. The primers SlDCL1IRDNA_F, which contains *Sal*I and *Pst*I sites at its 5′ end, and SlDCL1IRDNA_R were used to amplify the promoter sequence. Primers SlDCL1IRRNA_F and SlDCL1IRRNA_R, which contains *Hin*dIII*/Eco*RI sites at its 5′ end, were used to amplify an overlapping fragment that contained the cDNA sequence. Then the 674bp product was assembled by using the amplified fragments as a template for PCR with primer pair SlDCL1IRDNA_F and SlDCL1IRRNA_R. The amplified assembled fragment was restricted with either *Pst*I/*Eco*RI or *Sal*I/*Hin*dIII and cloned in sense and antisense orientation, respectively, around the first intron of the *Arabidopsis AKT1* gene to generate max2intpFLAP-SlDCL1IR. Following sequence validation, the max2intpFLAP-SlDCL1IR *Xho*I fragment was mobilized into the *Xho*I site of the OP-TATA-BJ36 shuttle vector between an *OP* array (Moore *et al.*, 1998) and *Agrobacterium tumefaciens* octopine synthase terminator (OCS) to generate OP:SlDCL1IR. Following orientation validation, the *Not*I fragment of the OP:SlDCL1IR vector was mobilized into the binary vector pART27 to generate pART27-OP:SlDCL1IR.

### Transformation of tomato plants

The binary vector pART27-OP:SlDCL1IR was transformed into tomato cv. M82 as described previously (Hendelman *et al.*, 2013*a*). Transgenic progeny were selected by germinating sterilized seeds on selective medium [1× Murashige and Skoog (MS) medium, 3% (w/v) sucrose, 100mg l^–1^ kanamycin], where only transgenic seedlings developed a branched root system. Further validation was performed by PCR of genomic DNA with the primer pair pFlap_intron_F and OCS_rev to detect the *OP:SlDCL1IR* transgene.

### Total RNA extraction and small RNA gel-blot analysis

Total RNA was isolated from tomato tissues as described previously (Hendelman *et al.*, 2013*a*). Blot hybridization analysis was performed according to Stav *et al.* (2010). Radiolabelled probes were made by 5′-end-labelling DNA oligonucleotides complementary to the corresponding small RNA sequences (probe sequences are listed in Supplementary Table S1 available at *JXB* online) with [γ-^32^P]ATP by means of T4 polynucleotide kinase (New England Biolabs, Ipswich, MA, USA). Blots were pre-hybridized and hybridized with EZ-hybridization solution (Biological Industries, Bet-Haemek, Israel). Hybridization was performed at 40 °C overnight. Blots were washed three times at 50 °C with washing buffer (2× SSC, 0.1% SDS) and autoradiographed using a phosphoimager (Fuji).

### Real-time quantitative PCR (qRT-PCR) analyses

Total RNA was extracted from the indicated tomato tissues as described above. DNase treatment, concentration determination, preparation of cDNA from 1 μg of total RNA, and real-time quantitative PCR were performed as described in Hendelman *et al.* (2013*b*). The relative expression levels of all transcripts were calculated using the two standard curve method normalized to *TIP41* or *CAC* as reference genes.

### Small RNA libraries constructions, sequencing, and bioinformatic analyses

Total RNA was extracted from shoots of control (*35S:LhG4*) and *SlDCL1*-silenced (*35S>>SlDCL1*) seedlings 14 d after sowing (two biological replicates per genotype combined from 12 seedlings each) using Bio-TRI RNA reagent (Bio-Lab, Jerusalem, Israel) according to the manufacturer’s protocol. Extracted RNA samples were used to construct two control and two *SlDCL1*-silenced small RNA libraries (Supplementary Table S2 available at *JXB* online) using the TruSeq small RNA kit (Illumina, San Diego, CA, USA) as described by the manufacturer, and submitted to sequencing on an Illumina HiSeq 2000 analyser. Sequencing was performed by Illumina. The data for this article have been deposited at the National Center for Biotechnology Information Gene Expression Omnibus (http://www.ncbi.nlm.nih.gov/geo/) under accession number GSE51959. The small RNA sequences from all sequenced libraries were parsed, mapped, quantified, and managed using The Cache ASsisted Hash Search with Xor logic (CASHX) Pipeline (release 2.3) (Fahlgren *et al.*, 2009). All reads were mapped to the tomato genome (SGN release version SL2.40; http://solgenomics.net/organism/Solanum_lycopersicum/genome) and perfectly matching reads in the size range of 18–26 nt were retained. The small RNA CASHX database was annotated with the Repeats Master version 5.0 (ftp://ftp.solgenomics.net/tomato_genome/repeats/), Plant snoRNAs Database (version 1.2; http://bioinf.scri.sari.ac.uk/cgi-bin/plant_snorna/home), SILVA rRNA database (http://www.arb-silva.de/), miRbase (release 19, http://www.mirbase.org/), and the tomato genome annotation (SGN release version ITAG2.3; ftp://ftp.solgenomics.net/tomato_genome/annotation/ITAG2.3_release/). After annotation, only ‘clean’ reads not matching tRNAs, rRNAs, small nuclear RNAs (snRNAs), or small nucleolar RNAs (snoRNAs) were used for further analysis. For all small RNA data analyses, clean reads from both biological replicates were used. The differential expression between control and *SlDCL1*-silenced clean small RNAs (two biological replicates per genotype) was performed by the analysis of digital gene expression data in the R (EdgeR) package (version 3.07) (Robinson *et al.*, 2010). By using this package, first low count reads were removed in each library by retaining reads with at least 1 count per million in at least three samples. Then normalization factors were calculated, effective library sizes were estimated by using normalization factors, fitted according to the Negative Binomial model, estimates of common dispersions were made, and differential expression tests were performed. The resultant *P*-values were adjusted for false discovery rate (FDR) and only adjusted *P*-values <0.05 were considered statistically significant. Cluster analysis of clean reads against the tomato genome was performed using the ShortStack software tool (version 0.4.1) (Axtell, 2013*b*) with options -mindepth=10 and -inv. The Inv file was generated from the tomato SL2.40 genome by einverted (EMBOSS 6.3.1) with option -maxrepeat 10000. Clean small RNAs from both biological replicates of each genotype were used as input to the ShortStack pipeline.

### Carotenoid determination in petals

Total pigments from petals at anthesis (~100mg) were extracted with acetone. Acetone samples were dried under a stream of N_2_ and dissolved in 450 μl of ethanol. Then, 50 μl of 60% KOH (w/v) were added and samples were incubated in the dark for 16h at 4 °C for saponification. Saponified samples were mixed gently with 0.5ml of diethyl ether and 0.1ml of 12% (w/v) NaCl/H_2_O. The upper phase was collected and dried under a stream of N_2_ and dissolved in acetone for further analysis. Total carotenoid content was determined spectroscopically at 440nm using an extinction coefficient of 2400 (Schiedt and Liaaen-Jensen, 1995). For identification, carotenoids were separated by reverse-phase high performance liquid chromatography (RP-HPLC) using a Waters 600 system (Waters, Milford, MA, USA) and a Spherisorb ODS2 C18 column (silica 5 μm, 3.2 mm×250mm) (Phenomenex, Torrance, CA, USA). The mobile phase consisted of acetonitrile:water (9:1; A) and ethylacetate (B), at a constant flow rate of 1.6ml min^–1^. The gradient was: 100% to 80% A during 8min; 80% to 65% A during 4min; followed by 65% to 45% A during 14min; and a final segment of 7min at 100% B. Spectra at the wavelength range of 250–700nm of eluting HPLC solvent were recorded and absorption peaks were detected. Carotenoids were identified by their absorption spectra and retention time.

## Results

### Generation of a reporter line that is able to silence the tomato *DCL1* homologue upon *trans*-activation

The full-length sequence of the tomato *DCL1* homologue was determined (*SlDCL1*; GenBank accession no. JX962774). Sequence analysis suggested that it is targeted by sly-miR162, similar to *AtDCL1* mRNA (Xie *et al.*, 2003), and encodes a DCL1-like protein (Supplementary Fig. S1A available at *JXB* online). Analysis of published RNA sequencing data indicated that *SlDCL1* is expressed at similar levels in vegetative and reproductive organs, with maximum expression in the root and minimum expression in breaking fruit (Tomato Genome Consortium, 2012) (Supplementary Fig. S1B available at *JXB* online).

**Fig. 1.**
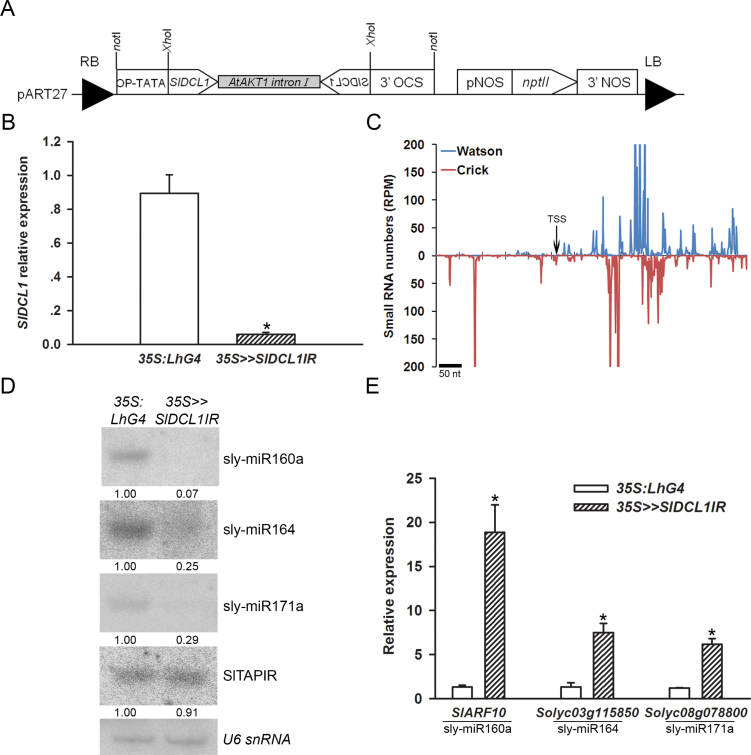
Molecular characterization of 35S>>*SlDCL1IR* plants. (A) Schematic representation of the binary responder *SlDCL1* RNAi (pART27-OP:SlDCL1IR) construct. (B) Quantitative RT-PCR analysis of *SlDCL1* transcript in 35S>>*SlDCL1IR* seedlings 28 d after sowing. Primers were designed around the miR162 complementary site, and *TIP41* expression values were used for normalization. Data are means ±SD of three independent biological repeats, each measured in triplicate. An asterisk indicates a significant difference as determined by Student’s *t*-test (*P* ≤ 0.01). (C) Schematic of siRNA accumulation at the *SlDCL1IR* RNAi sequence. The siRNA sequences were determined by deep sequencing of the seedling RNA used for qRT-PCR analysis in B. The normalized abundances of siRNAs were plotted relative to the sequence used for RNAi (Supplementary Table S1 available at *JXB* online) as a function of the positions of their 5′ ends. Maximum normalized reads per million (RPM) were set at 200/–200 to enable the visualization of relatively low abundance siRNAs. TSS, transcription start site as determined by RACE. (D) RNA gel-blot analysis of small RNAs in the RNA samples analysed in B. The total RNA (2.5 μg) was hybridized with the indicated small RNA antisense probe. An antisense probe for *U6* snRNA served as a loading control. Small RNA expression was normalized to *U6* snRNA, and levels are indicated below each panel. (E) qRT-PCR analysis of miRNA target transcripts in the RNA samples analysed in B. The corresponding miRNA is shown below each target mRNA. *TIP41* expression values were used for normalization. Error bars indicate ±SD of three biological replicates, each measured in triplicate. Asterisks indicate significant difference as determined by Student’s *t*-test (*P* ≤ 0.01).

**Fig. 2.**
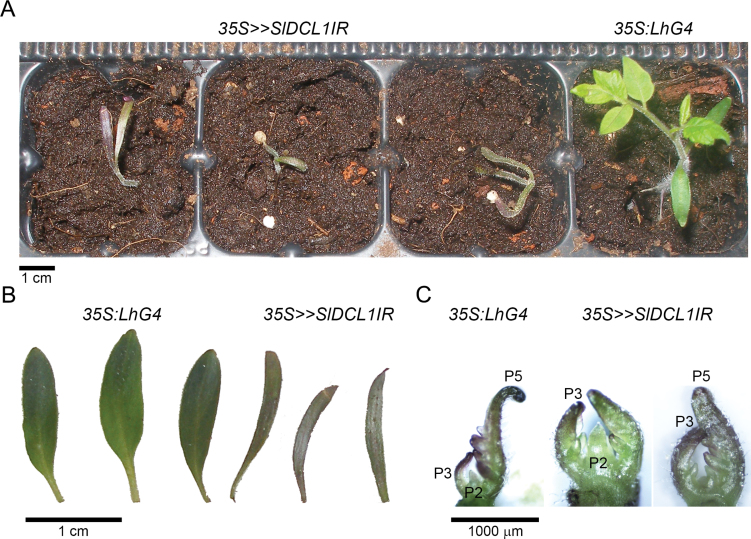
Phenotypic characterization of *35S>>SlDCL1IR* seedlings. (A–C) Phenotypes of control (*35S:LhG4*) and *35S>>SlDCL1IR* plants. (A) Seedlings at 28 d after sowing. (B) A magnified view of cotyledons from the seedlings in A. (C) A magnified view of shoot apices from the seedlings in A.

In the absence of a tomato *dcl1* mutant, its function was addressed by silencing it in transgenic tomato. However, plant *dcl1* loss-of-function mutants are likely to be either lethal or arrested during early development (Errampalli *et al.*, 1991; Castle *et al.*, 1993; Liu *et al.*, 2005). To overcome this problem, a *trans*-activated RNAi was employed via the OP/LhG4 transcription factor system. A responder RNAi construct (*OP:SlDCL1IR*) that spanned the transcriptional start site of *SlDCL1* was constructed with the intention of improving knockdown efficiency by invoking post-transcriptional as well as transcriptional silencing of *SlDCL1* (Fig. 1A; Supplementary Fig. S1A available at *JXB* online). Following *OP:SlDCL1IR* transformation into M82 tomato and characterization of regenerated transgenic responder plants, the *OP:SlDCL1IR-13* responder line (hereafter *OP:SlDCL1IR*) was selected for further analysis because *SlDCL1* was almost completely silenced (~5% of control plants) in its *trans-*activated F_1_ progeny (*35S*>>*SlDCL1IR*) (Fig. 1B). The strong silencing was associated with the accumulation of siRNAs that matched *SlDCL1* mRNA as well as its putative promoter (Fig. 1C), demonstrating the functionality of the RNAi construct and the usefulness of *trans*-activation for *DCL1* silencing. In agreement with DCL1 being the primary producer of canonical miRNAs (Park *et al.*, 2002; Kurihara and Watanabe, 2004), northern blots of *35S>>SlDCL1IR* seedlings revealed decreased levels of several known tomato miRNAs, but no significant change in the levels of type III fold-back transposon anionic peroxidase inverted repeat (TAPIR)-derived heterochromatic siRNA (Moxon *et al.*, 2008) (Fig. 1D). Consistent with the down-regulation of miRNAs, corresponding target mRNA levels increased significantly (Fig. 1E). In accordance with the perturbation of their miRNA pathway, the *35S>>SlDCL1IR* seedlings could only germinate in tissue culture, had narrow cotyledons, and their development was arrested at the first to third true leaf primordium stage (Fig. 2).

### Small RNAs that depend on *SlDCL1* for their biogenesis

To determine the effect of *SlDCL1* silencing on global small RNA metabolism in tomato, the small RNA populations of control and *35S>>SlDCL1IR* seedlings were compared using Illumina sequencing by synthesis technology. Two small RNA libraries, representing biological replicates, were prepared and sequenced from each genotype, generating >19 million raw reads in total (Supplementary Table S2 available at *JXB* online). For the analyses presented here, only sequences that were 18–26 nt in length were used. Depending on the library, read representation was between 6 and 4 million, from which ~70% matched the published tomato genome. Around 30% of the matched reads originated from tRNA, rRNA, snRNA, and snoRNA sequences. The remaining genome-matched reads (referred to as clean reads hereafter) were used for further analysis and represented ~1 million distinct tomato small RNA sequences (Supplementary Table S2 available at *JXB* online). The abundance of small RNAs was calculated as reads per million (RPM). After processing, clean small RNAs were annotated as described in the Materials and methods (Supplementary Table S3 available at *JXB* online).

**Fig. 3.**
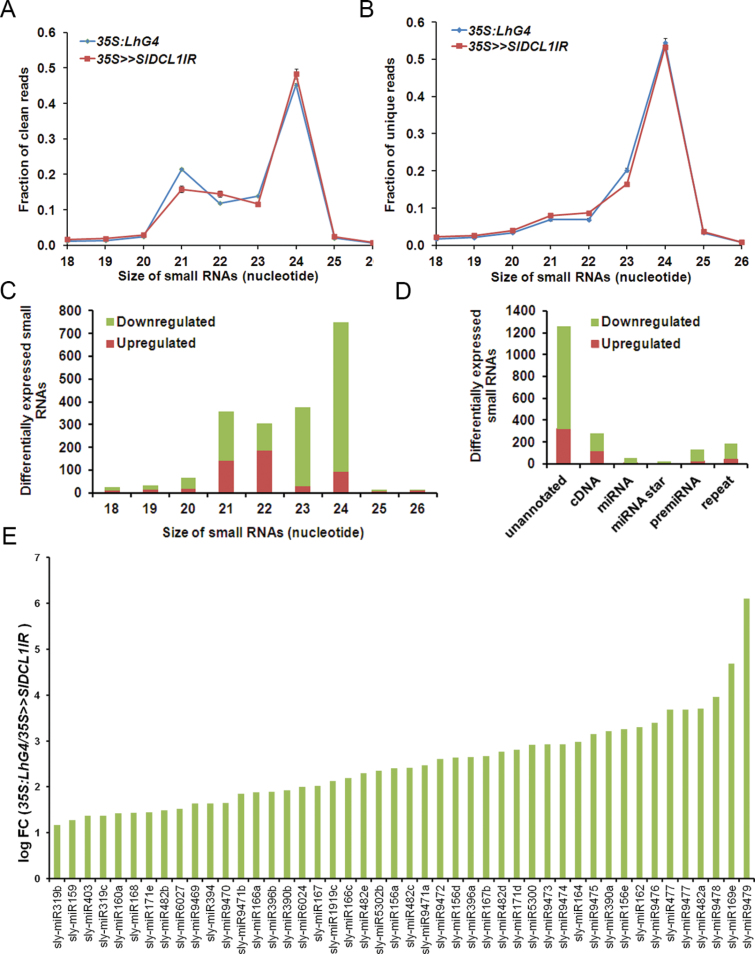
Characterization of small RNAs from control (*35S:LhG4*) and *35S>>SlDCL1IR* seedlings. (A, B) Size distribution of small RNAs in the indicated genotypes. The distributions of total (A) and unique (B) small RNAs are shown. Data are means ±SD of two biological replicates. (C–E) Characterization of differentially expressed small RNAs. (C) Size distribution of differentially expressed small RNAs. (D) Features of genomic loci that generate the small RNAs shown in C. (E) Change in miRNA accumulation upon *SlDCL1* silencing.

Consistent with the distribution of small RNA sizes that is typically observed in rice and *Arabidopsis* (Kasschau *et al.*, 2007; Wu *et al.*, 2010), in *35S:LhG4*, the 24-nt small RNAs accounted for 45% of the population, making them the most abundant size class, with 21-nt small RNAs forming a secondary peak (Fig. 3A). Compared with *35S:LhG4* control seedlings, in *35S>>SlDCL1IR*, the 21-nt size class was under-represented, whereas the 22-nt size class was over-represented (Fig. 3A), but a significant change in diversity was not observed (Fig. 3B). The question then was which tomato small RNAs require *SlDCL1* for their biogenesis. EdgeR and ShortStack analyses were employed to identify the small RNAs and their producing loci, respectively, that show significantly modified expression on *SlDCL1* down-regulation (Robinson *et al.*, 2010; Axtell, 2013*b*). First, to identify and annotate the wild-type tomato small RNA clusters, the *35S:LhG4* clean small RNAs were subjected to ShortStack analysis. Then, the expression of *35S>>SlDCL1IR* clean small RNAs from identified wild-type clusters was quantified and the clusters were re-annotated accordingly. ShortStack analysis of control clean small RNAs identified 128 621 small RNA-producing clusters, of which 92 were annotated as *MIRNA*-derived clusters (Supplementary Table S4 available at *JXB* online). Since DCL1 is known to catalyse the formation of predominantly 21-nt RNAs (Park *et al.*, 2002; Reinhart *et al.*, 2002; Qi *et al.*, 2005), the analysis was focused on the clusters that contained significant numbers (≥25 RPM) of 21-nt small RNAs and are therefore more likely to involve SlDCL1 in their biogenesis. Only 995 clusters fulfilled this criterion, and 132 of these showed at least a 2-fold change in their 21-nt small RNA numbers upon *SlDCL1* silencing (hereafter termed DEclusters). Eighty-nine DEclusters were underexpressed in *35S>>SlDCL1IR* relative to controls (Supplementary Table S5 available at *JXB* online). Cluster_61425, which derived from the *MIR395a* locus and contained ~30-fold fewer 21-nt small RNAs in *35S>>SlDCL1IR*, showed the strongest decrease. As expected, MIRNA-derived small RNAs were almost exclusively down-regulated in *35S>>SlDCL1IR* seedlings, and *MIRNA*-encoding loci comprised the largest group (61%, 32/89) of underexpressed DEclusters (Supplementary Table S5 available at *JXB* online). The results of the ShortStack analysis also indicated that several miRBase tomato miRNAs were not the most abundant small RNAs from their hairpin precursors and corresponded either to the predicted miRNA* [sly-miR6027 (Cluster_134922), sly-miR1919a-c (1892963), sly-miR5301 (Cluster_93957)] or to an undefined pre-miRNA position [sly-miR5302 (Cluster_93957)]. In the case of sly-miR5301, the predicted miRNA was found to be a close homologue of miR482. EdgeR analysis identified 2.5% (1421 of 56 052) and 0.9% (494 of 56 052) of the relatively abundant clean small RNAs (at least 1 RPM in three libraries), that had at least 2-fold fewer or more reads (FDR ≤0.05), respectively, in *35S>>SlDCL1IR* relative to the control (Supplementary Table S6 available at *JXB* online). Among the down-regulated small RNAs, many were 21–22 nt in length originating from *MIRNA* genes, but also small RNAs 23–24 nt in length, which derived from non-annotated genomic loci and not likely to be produced by SlDCL1 (Qi *et al.*, 2005), were present (Fig. 3C, D). In contrast, among the up-regulated small RNAs, most were 21–22 nt in length, derived from non-annotated genomic loci (Fig. 3C, D). In agreement with the ShortStack analysis, edgeR analysis indicated the significant down-regulation of 47 miRNAs in *35S>>SlDCL1IR* relative to controls (Supplementary Table S6 available at *JXB* online). Annotation of these miRNAs indicated that 14 were identical to miRBase, 16 were homologues of known miRNAs, and 17 represented novel miRNAs. The degree of miRNA down-regulation was 4-fold on average, but varied considerably between miRNAs, indicating differential *SlDCL1* requirement for their biogenesis (Fig. 3E).

**Fig. 4.**
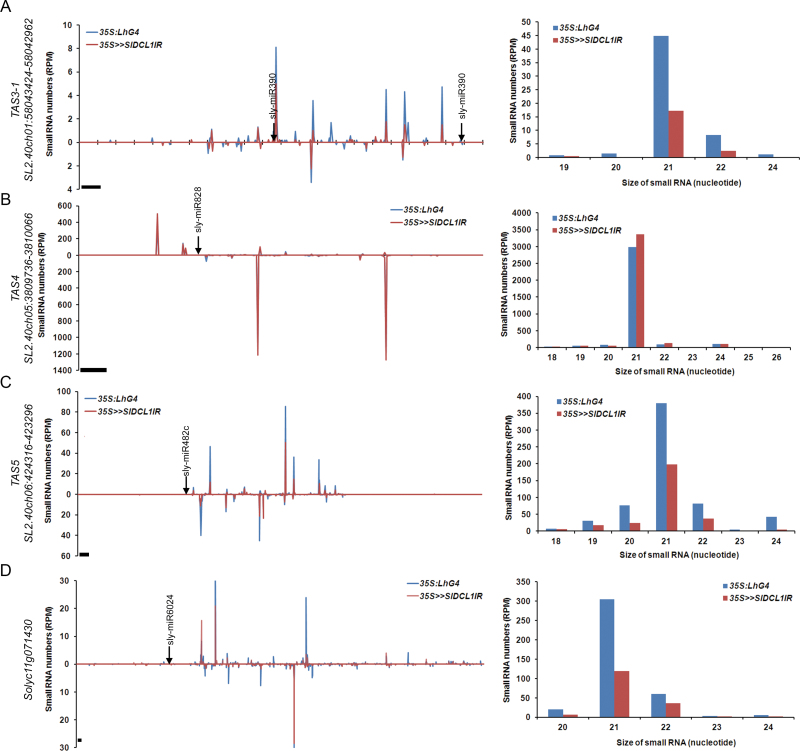
Effect of *SlDCL1* silencing on phased siRNA accumulation. Schematic of *35S>>SlDCL1IR* and control siRNA accumulation at indicated *TAS* (A–C) and *R* genes (D). The normalized abundances of siRNAs were plotted relative to their mRNA sequence as a function of the positions of their 5′ ends. Values above and below zero indicate siRNA matching of the plus and minus strand, respectively. The corresponding miRNA cleavage site is indicated. Bars=21 nt. Size distribution of total gene-derived siRNAs is shown on the right. Data are means of two biological replicates.

In tomato, sly-miR390- and sly-miR482c-guided cleavage sets the phase for the biogenesis of *trans*-acting siRNA gene 3 (*TAS3*)-derived and *TAS5*-derived ta-siRNAs, respectively (Li *et al.*, 2012*a*). EdgeR analysis suggested that sly-miR390 and sly-miR482c are significantly down-regulated, by 3.9- and 5.4-fold, respectively, in *35S>>SlDCL1IR* seedlings (Fig. 3E). Consistent with this, the present analysis revealed significantly fewer *TAS3-1* and *TAS5* ta-siRNAs in *35S>>SlDCL1IR* seedlings, thus confirming the requirement of *SlDCL1* for ta-siRNA biogenesis (Fig. 4A, C). In contrast, a slight increase, rather than the expected decrease, was observed in the numbers of *TAS4*-derived (Cluster 99854) ta-siRNAs (Fig. 4B). Since the expression of *TAS4* is induced by different stresses (Hsieh *et al.*, 2009), a possible explanation is that an increase in *TAS4* transcription occurred in the stressed *35S>>SlDCL1IR* seedlings.

Except for ta-siRNAs, which depend on initial cleavage by miRNA for their biogenesis (Allen *et al.*, 2005), cleavage by 22-nt miRNAs was found to set up the production of secondary 21-nt siRNAs from various target RNAs (Chen *et al.*, 2010; Cuperus *et al.*, 2010). In tomato and *Medicago*, such a mechanism was suggested to trigger the production of phased secondary 21-nt siRNAs from nucleotide-binding site (NBS)–leucine-rich repeat (LRR) *R* genes, thus preventing their unregulated expression (Li *et al.*, 2010; Zhai *et al.*, 2011; Shivaprasad *et al.*, 2012). Accordingly, most (78%, 14/18) of the gene-derived DEclusters contained 21-nt siRNAs that matched NBS–LRR-encoding loci (Supplementary Table S5 available at *JXB* online). The 21-nt small RNA numbers from this DEcluster set were reduced by 2- to 5.3-fold upon *SlDCL1* silencing, probably as a result of lower levels of the miRNA that triggers their biogenesis. An example of an NBS–LRR gene-derived DEcluster (Cluster_146573), which is predicted to be triggered by sly-miR6024-mediated cleavage, is shown in Fig. 4D. Indeed, in *35S>>SlDCL1IR* seedlings, sly-miR6024 was down-regulated 4-fold and *Solyc11g071430*-derived 21-nt phased siRNA numbers decreased by 2.6-fold. The rest of the down-regulated gene-derived DEclusters were generated from genes with unknown functions. In addition, the analysis identified 13 repeat-associated DEclusters that were down-regulated upon *SlDCL1* silencing, suggesting its involvement in the biogenesis of siRNAs from these loci (Supplementary Table S5 available at *JXB* online).

### *SlDCL1* silencing induces the up-regulation of certain *SlDCL2* genes

**Fig. 5.**
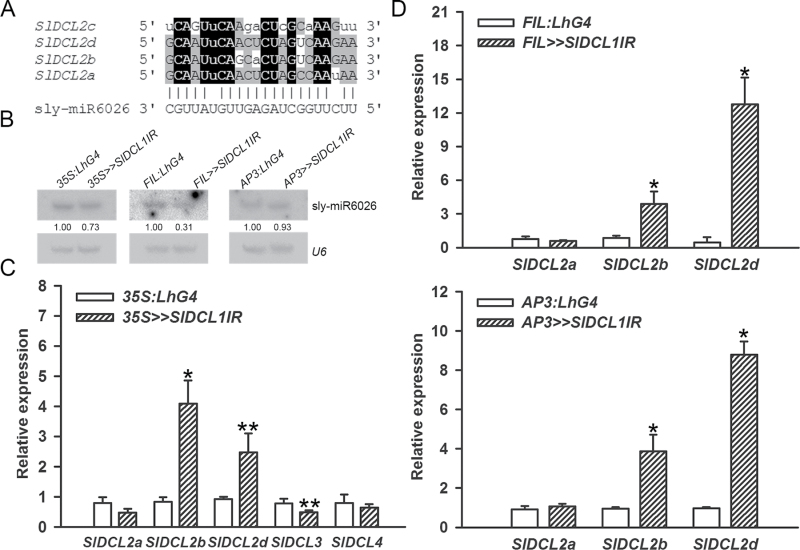
Certain *SlDCL2* paralogues are up-regulated upon *SlDCL1* silencing. Sequence alignment between *SlDCL2* genes (A) predicted to serve as sly-miR6026 targets. Identical nucleotides in all or at least three sequences are shaded in black and grey, respectively. Watson–Crick pairing with sly-miR6026 is shown below. Mismatched nucleotides are in lower case. (B) RNA gel-blot analysis of sly-miR6026 in the indicated genotypes from seedlings 28 d after sowing, leaf P6-7 primordia, and flower petals in 10mm buds (left to right). Total RNA (5 μg) from the indicated tissues was hybridized with sly-miR6026 antisense probe. Sly-miR6026 expression was normalized to *U6* snRNA and levels are indicated below each panel. (C, D) qRT-PCR analysis of the expression levels of the indicated *SlDCL* genes in the RNA samples analysed in B: seedlings 28 d after sowing (C), leaf P6-7 primordia (D top), and flower petals in 10mm buds (D bottom). Error bars indicate ±SD of three biological replicates, each measured in triplicate. Single (*P* ≤ 0.01) and double (*P* ≤ 0.05) asterisks indicate significant difference as determined by Student’s *t*-test.

It has been suggested that soybean and *Medicago DCL2* mRNAs are targeted by the 22-nt miR1515 and miR1507, respectively, triggering the generation of 21-nt secondary phased siRNAs (Li *et al.*, 2010; Zhai *et al.*, 2011). Tomato has four DCL2 genes (*SlDCL2a–d*), three of which (*SlDCL2b–d*) are clustered on chromosome 11 (SL2.40ch11:2690601–2724508) (Bai *et al.*, 2012). Similar to *Medicago DCL2*, ShortStack analysis identified small RNA clusters that were derived from *SlDCL2a* (clusters 78419–78430), *SlDCL2b* (clusters 136462–136472), *SlDCL2c* (clusters 136447–136454), and *SlDCL2d* (clusters 136455–136460). These clusters were almost all (34/35) dominated by 21-nt small RNAs (DicerCall=21) that in a few cases were significantly phased (Supplementary Table S5, Fig. S2 available at *JXB* online). In addition, a legitimate target site for the 22-nt sly-miR6026 was predicted in the 5′ UTRs of *SlDCL2a*, *b*, and *d* but not in *SlDCL2c*, which contains a different sequence instead (Fig. 5A). Cleavage by 22-nt miRNAs has been shown to induce the biogenesis of 21-nt secondary siRNAs (Cuperus *et al.*, 2010), raising the possibility that sly-miR6026 acts as a trigger for siRNA biogenesis from *SlDCL2a*, *b*, and *d*. In *Medicago*, miR1507 has been suggested to target *DCL2* as well as NBS–LRR genes (Zhai *et al.*, 2011). Consistent with this, sly-miR6026 has been predicted to target the *Tobacco mosaic virus* resistance gene of tomato (*TM2*, *Solyc09g018220*) (Li *et al.*, 2012*b*), and ShortStack analysis identified 21-nt small RNA clusters that derived from it (clusters 113162 and 113163) (Supplementary Table S5 available at *JXB* online), suggesting that similar to miR1507 in *Medicago*, in tomato, sly-miR6026-guided cleavage of *SlDCL2a*, *b*, and *d* as well as the NBS–LRR *TM2* gene triggers secondary siRNA production. RNA gel blots detected a slight decrease in sly-miR6026 upon global and tissue-specific (see below) *SlDCL1* silencing (Fig. 5B). However, the present analysis indicated that a significant increase, rather than a decrease, in the numbers of *SlDCL2a*, *b*, and *d*-derived small RNAs had occurred in *35S>>SlDCL1IR* seedlings (Supplementary Table S5, Fig. S2 available at *JXB* online). The most significant increase was in *SlDCL2b* (DEclusters 136464–136470, average of ~4.73±0.97-fold) and *SlDCL2d* (DEclusters 136455–136458, average of ~3.13±1.02-fold). Possible explanations include an increase in the expression of either *SlDCL4*, which is required for 21-nt phased siRNA biogenesis (Yifhar *et al.*, 2012), or *SlDCL2b* and *d*, whose transcripts may serve as substrates for sly-miR6026-mediated cleavage. qRT-PCR analysis of these genes in different *SlDCL1*-depleted tissues confirmed the significant increase in *SlDCL2b* and *d* accumulation, whereas accumulation of *SlDCL2a*, *SlDCL3*, and *SlDCL4* was not significantly changed (Fig. 5C, D). Since DCL2 has been shown to be required for the formation of 22-nt siRNAs (Xie *et al.*, 2004; Gasciolli *et al.*, 2005; Deleris *et al.*, 2006), the up-regulation of *SlDCL2b* and *d* in *35S>>SlDCL1IR* seedlings might explain the global increase in 22-nt numbers (Fig. 3A) and the majority (43%) of the 22-nt up-regulated DEclusters (Supplementary Table S5 available at *JXB* online).

### Depletion of *SlDCL1* from developing leaf primordium inhibits blade expansion

**Fig. 6.**
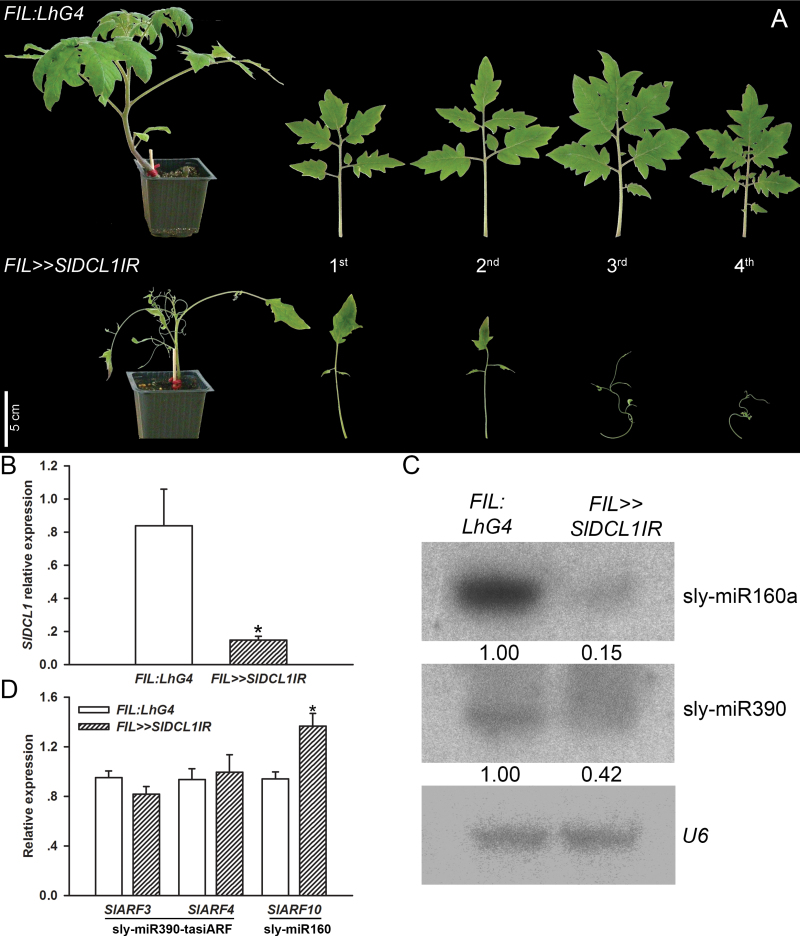
Characterization of *SlDCL1*-depleted leaves. (A) Comparison of whole M82 tomato plants 42 d after sowing and representative leaves (from left to right) between control (*FIL:LhG4*) and *FIL>>SlDCL1IR*. Scale bar=5cm. (B) qRT-PCR analysis of *SlDCL1* transcript levels in *FIL:LhG4* and *FIL>>SlDCL1IR* P5 leaf primordia. (C) RNA gel-blot analysis of miRNAs in the RNA samples analysed in B. Total RNA (2 μg) was hybridized with the indicated miRNA antisense probe. Expression levels were determined relative to the control after normalization to *U6* and are indicated below each panel. (D) qRT-PCR analysis of miRNA target transcripts in the RNA samples analysed in B. The corresponding miRNA is shown below each target mRNA. (B, D) *CAC* expression values were used for normalization. Error bars indicate ±SD of three biological replicates, each measured in triplicate. Asterisks indicate significant difference as determined by Student’s *t*-test (*P* ≤ 0.01).

Constitutive *SlDCL1* silencing induced seedling lethality before lateral organs could develop (Fig. 2). However, tissue-specific driver lines allowed investigation of the requirement of *SlDCL1* of leaf and flower development. In tomato, the *Filamentous Flower* (*FIL*) promoter drives strong expression throughout young leaf primordia soon after they initiate from the shoot apical meristem (SAM), but not while they are in the SAM (Lifschitz *et al.*, 2006; Shani *et al.*, 2009). Thus, leaf-specific *SlDCL1* silencing was induced by crossing the *OP:SlDCL1IR* responder line with the *FIL:LhG4* driver line. As expected, germination of *FIL>>SlDCL1IR* progeny seedlings was normal (Supplementary Fig. S3 available at *JXB* online). In addition, relatively subtle phenotypes were observed in the first two leaves. These leaves contained one instead of two pairs of primary leaflets, which were smaller than controls, and had a morphologically distinct terminal leaflet blade (Fig. 6A). More severe phenotypes were observed in later formed leaves. Compared with *FIL:LhG4* control leaves, these leaves were much smaller, and had wavy rachises that supported almost bladeless primary and terminal leaflets (Fig. 6A). As the plants matured, their cotyledons became abnormally elongated. In addition, precocious outgrowth of cotyledonary as well as juvenile axillary meristems was observed, suggesting loss of apical dominance in these plants (Supplementary Fig. S3 available at *JXB *online). The bladeless *FIL>>SlDCL1IR* leaf phenotype was reminiscent of the later formed wiry leaves of TAS3 pathway mutants that overaccumulate *Auxin Response Factor 3* (*SlARF3*) and *SlARF4*, due to reduced levels of ta-siARF (Yifhar *et al.*, 2012). Reduced tomato leaf lamina outgrowth has also been associated with ectopic accumulation of miR160-resistant *SlARF10* (Hendelman *et al.*, 2012). Molecular analysis of young leaf primordia confirmed the silencing of *SlDCL1* (Fig. 6B) and, accordingly, revealed reduced accumulation of sly-miR160a and sly-miR390, which is required for ta-siARF biogenesis (Fig. 6C) (Yifhar *et al.*, 2012). Nevertheless, significant up-regulation of *SlARF3*, *SlARF4*, and *SlARF10* was not detected in these primordia, most probably due to insufficient down-regulation of sly-miR390 and sly-miR160a, respectively (Fig. 6D).

### Partial depletion of *SlDCL1* from petals results in lower accumulation of carotenoids

**Fig. 7.**
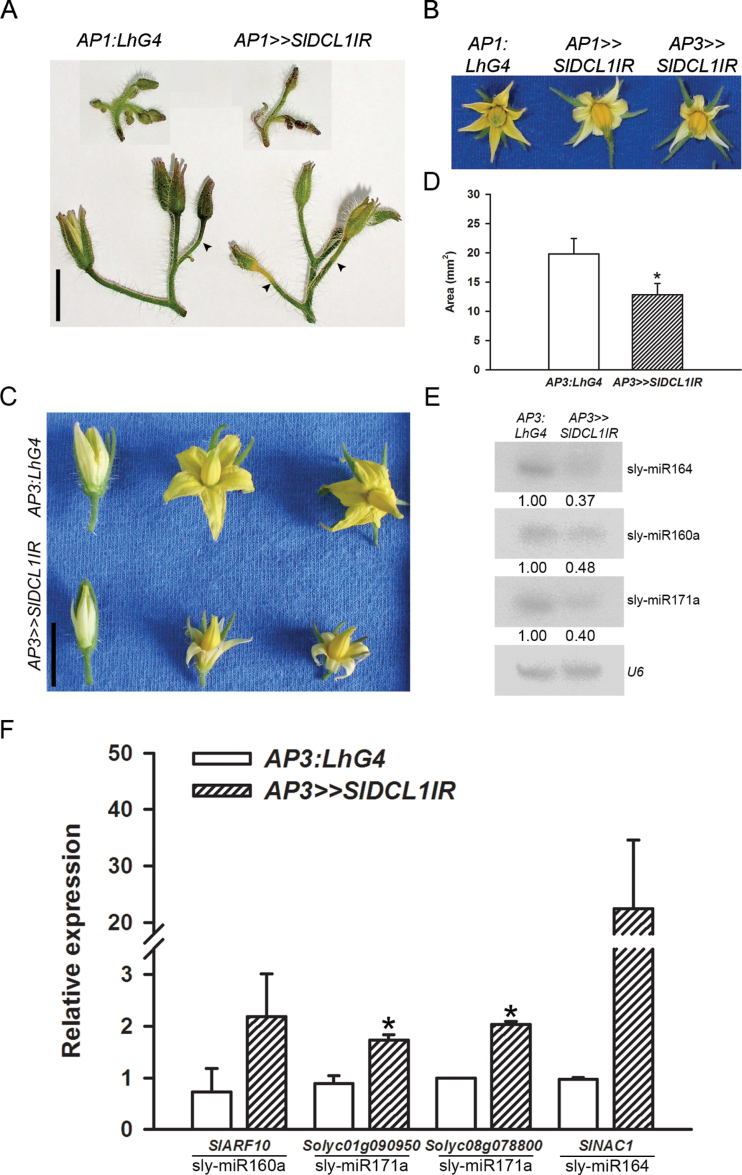
Characterization of *SlDCL1*-depleted flowers. (A) Phenotype of representative control (*AP1:LhG4*) and *AP1>>SlDCL1IR* inflorescences bearing young (top) and mature (bottom) buds. Arrowheads mark the abscission zone. Scale bars=1cm. (B) Representative flowers of the indicated genotypes at anthesis. (C) Representative flowers of the indicated genotypes at different developmental stages: 2 d before anthesis, anthesis, and 1 d post-anthesis (left to right). (D) Quantitation of petals area at anthesis in control and *AP3>>SlDCL1IR*. Error bars represent ± SD (*n*=54). (E) RNA gel-blot analysis of miRNAs in petals from 10mm buds of the indicated genotypes. Total RNA (5 μg) was hybridized with the corresponding miRNA antisense probe. Expression levels were determined relative to the control after normalization to U6 and are indicated below each panel. (F) qRT-PCR analysis of miRNA target transcripts in the RNA samples analysed in E. The corresponding miRNA is shown below each target mRNA. *CAC* expression values were used for normalization. Error bars indicate ±SD of three biological replicates, each measured in triplicate. (D, F) Asterisks indicate significant difference as determined by Student’s *t*-test (*P* ≤ 0.01).

The flower-specific *AP1:LhG4* driver line drives expression throughout the young floral primordia and, as the buds mature, its activity is mainly confined to developing sepals and petals. The *AP3:LhG4* driver line drives expression throughout the developing petals, in the stamen vasculature, and on the abaxial side of anthers (Hendelman *et al.*, 2013*a*). Hence, flower-specific *SlDCL1* silencing was induced by crossing the *OP:SlDCL1IR* responder line to these driver lines. Both *AP1>>SlDCL1IR* and *AP3>>SlDCL1IR* plants showed a wild-type phenotype during vegetative growth (data not shown). Moreover, young *AP1>>SlDCL1IR* buds did not show any developmental aberrations despite containing only 40% of the wild-type *SlDCL1* levels (Supplementary Fig. S4A available at *JXB* online). However, most of the buds underwent premature senescence at 4–5mm in size and could not develop into mature flowers. The senescence initiated as yellowing of the receptacle area in one or more buds from the same inflorescence. This yellowing spread downward along the stack to the abscission zone and upward to the sepals, ultimately leading to complete drying of the bud (Fig. 7A). Only ~10% of the buds developed into mature flowers that produced pale petals (Fig. 7B). Interestingly, the *AP3>>SlDCL1IR* flowers produced similarly pale and slightly smaller petals (Fig. 7B–D). The composition of carotenoids in *AP3>>SlDCL1IR* petals was analysed to determine whether the pale phenotype is associated with quantitative or qualitative differences. While the carotenoid profile in *AP3>>SlDCL1IR* petals is relatively similar to that of the control, consisting of mainly neoxanthin and violaxanthin, the total level of carotenoids was reduced >2-fold (Table 1). Additional visual abnormalities were not observed in the *AP3>>SLDCL1IR* flowers (Fig. 7C). qRT-PCR analysis of *SlDCL1* levels in isolated petals, at the start of yellowing, revealed that it accumulated to only 25% of controls, indicating partial silencing of *SlDCL1* in the petals (Supplementary Fig. S4B available at *JXB* online). Accordingly, miRNA levels were down-regulated and corresponding target mRNAs were up-regulated in the silenced petals (Fig. 7E, F).

**Table 1.** *Carotenoid composition in control and* AP3>>SlDCL1IR *petals*

	*AP3:LhG4* ^*a*^	*AP3>>SlDCL1lR* ^*a*^
Neoxanthin	71.6±2.2	66.4±4.2
Violaxanthin	24.3±1.9	23.0±2.4
Antheraxanthin	1.2±0.5	1.4±0.4
Lutein	1.8±0.4	5.6±1.9
β-Carotene	1.1±0.4	3.7±0.5
Total^*b*^ (μg g fresh weight^–1^)	620.6±112.8	246.4±28.8

^*a*^ Numbers correspond to percentage of total carotenoids (*n*=4).

^*b*^ All isomers.

## Discussion

As part of the effort to unveil the roles of miRNA regulation in tomato development, transgenic tomato responder lines were previously generated that, by *trans*-activation, can express the viral silencing suppressors *Tomato bushy stunt virus* P19 and *Beet western yellows virus* P0, thereby perturbing the miRNA pathway in the activated plants (Stav *et al.*, 2010; Hendelman *et al.*, 2013*a*). However, because P19 binds siRNA duplexes and P0 can lead to the decay of various ARGONAUTES, their transgenic expression may interfere with endogenous siRNA pathways as well (Dunoyer *et al.*, 2004; Baumberger *et al.*, 2007). In the current study, the *OP:SlDCL1IR* tomato responder line was used; this line is a more specific miRNA-inhibiting tool that enables the silencing of *SlDCL1*, the tomato homologue of *DCL1*, by *trans*-activation. DCL1 has been found to be the predominant DCL involved in the biogenesis of canonical miRNAs, with no other DCL acting as its surrogate (Gasciolli *et al.*, 2005). Accordingly, *trans*-silencing of *SlDCL1* to 5% of its normal levels was associated with diminished miRNA levels, demonstrating that in tomato, like in *Arabidopsis* and rice, DCL1 is central for miRNA production.

The involvement of DCL1 in the biogenesis of certain siRNAs renders the criterion of *DCL1* dependence for accumulation insufficient to warrant annotation of a small RNA as an miRNA (Meyers *et al.*, 2008). ShortStack is a recently published stand-alone application that can predict and annotate plant miRNAs based on small RNA sequencing data (Axtell, 2013*b*). By combining ShortStack predictions and the *SlDCL1* dependence criterion, 33 new *SlDCL1*-dependent miRNAs were confidently discovered, 17 of which represent novel plant miRNAs. This method is suggested for reliable annotation of canonical miRNA in tomato. A total of 47 miRNAs that depend on *SlDCL1* for their biogenesis were identified in this study. These miRNAs were shown to be differentially reduced by *SlDCL1* silencing. This differential response ranged from a 2-fold to a 64-fold decrease and might indicate the *in vivo* cleavage efficiency and specificity of SlDCL1 for various miRNA precursors. Interestingly, sly-miR159a, which is encoded by a single precursor (SL2.40ch03:61786005–61786301), was reduced by only 2.4-fold in *35S>>SlDCL1IR* plants, indicating relatively efficient processing and low sensitivity to *SlDCL1* silencing. Indeed, the sly-miR159a precursor has been successfully used as a backbone for the expression of an artificial miRNA (amiR) precursor in transgenic tomato and it was more efficiently processed than sly-miR168a-based amiR (reduced by 2.7-fold in *35S>>SlDCL1IR*) (Vu *et al.*, 2013).

The present data revealed that *SlDCL1* silencing causes two significant but contrasting changes in the small RNA population of tomato seedlings. The *35S>>SlDCL1IR* seedlings exhibited a lower proportion of 21-nt small RNAs and a higher proportion of 22-nt small RNAs. The former was also observed in other *dcl1* mutants and can be attributed to a substantial reduction in 21-nt miRNAs which depend on DCL1 for their biogenesis, and phased siRNAs, which depend on initial miRNA cleavage for their production (Kasschau *et al.*, 2007; Shivaprasad *et al.*, 2012). Nevertheless, the increase in 22-nt small RNAs has not been observed in other *dcl1* mutants (Kasschau *et al.*, 2007; Wu *et al.*, 2010). In *Arabidopsis*, DCL2 has been shown to be required for the formation of 22-nt long siRNAs (Xie *et al.*, 2004; Gasciolli *et al.*, 2005; Deleris *et al.*, 2006). The tomato genome encodes four *DCL2* paralogues, three of which (*SlDCL2a*, *b*, and *d*) are clustered on chromosome 11 (Bai *et al.*, 2012). The results of the present study indicate that *SlDCl2b* and *SlDCl2d* are up-regulated upon *SlDCL1* silencing, suggesting that these paralogues are responsible for the observed increase in 22-nt small RNAs. Sly-miR6026, which is predicted to target *SlDCL2a*, *b*, and *d*, was not significantly down-regulated upon *SlDCL1* silencing. Consistent with this, *SlDCL2b*- and *SlDCL2d*-derived siRNAs increased rather than decreased and *SlDCl2a* was not up-regulated in *35S>>SlDCL1IR* seedlings. Therefore, the up-regulation of *SlDCl2b* and *SlDCl2d* is probably due to an increase in transcription rather than reduced targeting by sly-miR6026. One appealing explanation for this is the existence of a specific *SlDCl2b* and *SlDCl2d* miRNA-regulated transcriptional activator that is up-regulated upon *SlDCL1* silencing.

Consistent with the severe developmental phenotypes of known *dcl1* mutants (Schauer *et al.*, 2002; Liu *et al.*, 2005; Khraiwesh *et al.*, 2010), the *35S>>SlDCL1IR* seedlings were growth arrested at the first to third leaf stage, emphasizing the importance of miRNAs for tomato’s post-germination development. Nevertheless, the *trans*-activated RNAi strategy enabled, for the first time, *SlDCL1* silencing in a specific organ, without disturbing prior development. This enabled a direct unbiased assessment of *SlDCL1* functions in the silenced tissue. In addition, activation of the *OP:SlDCL1IR* construct by different promoters generated variable silencing strengths that were manifested by hypomorphic phenotypes. Reducing *SlDCL1* levels by 85% in the leaf primordia led to strong inhibition of blade outgrowth, but did not modify its abaxial–adaxial polarity, regulated in part by miR166 and miR390 (Chitwood *et al.*, 2007). Similarly, the hypomorphic *Arabidopsis* mutant *dcl1-9* (*caf*) produced polar leaves that were thinner than the wild type and ~10% of them did not develop any blade (Jacobsen *et al.*, 1999), together indicating that blade outgrowth is dependent on DCL1-mediated silencing in simple as in compound leaf species. Indeed, perturbation of the post-transcriptional regulation of *SlARF10*, and *SlARF3* and *SlARF4*, which are normally cleaved by sly-miR160a and tasiARF, respectively, has been shown to reduce blade outgrowth dramatically in tomato leaves (Hendelman *et al.*, 2012; Yifhar *et al.*, 2012). Surprisingly, none of these target genes was significantly up-regulated in *FIL>>SlDCL1IR* leaf primordia, suggesting that they are not the main cause for the bladeless phenotype. In addition, *FIL>>SlDCL1IR* plants also demonstrated loss of apical dominance. Since both blade outgrowth and apical dominance are regulated by auxin (Cline, 1997; Koenig *et al.*, 2009), disruption of its signalling pathway by *SlDCL1* silencing is likely to be a major cause for the observed *FIL>>SlDCL1IR* phenotypes.

The distinct yellow colour of tomato flowers is a result of accumulation of carotenoids, mainly the xanthophylls neoxanthin and violaxanthin (Hirschberg, 2001). A novel pale phenotype was observed in *AP1>>SlDCL1IR* and *AP3>>SlDCL1IR* petals with partially silenced *SlDCL1*. This phenotype was associated with a reduction of >2-fold in the total rather than an alteration in the ratio between the individual carotenoids. The exact reason for this is currently unknown. Nevertheless, by profiling *Arabidopsis dcl1* mutant embryos, it was shown that the two most up-regulated miRNA targets are responsible for a large part of the mutant embryo phenotype, thus proving the usefulness of *dcl1* mutants for revealing specific miRNA regulatory modules involved in development (Nodine and Bartel, 2010; Willmann *et al.*, 2011). The availability of a tomato *dcl1* mutant, as presented in the current study, now enables the use of a similar approach to elucidate the molecular bases of the observed phenotypes.

## Supplementary data

Supplementary data are available at *JXB* online.

Figure S1. Sequence analysis of the tomato DCL1 homologue.

Figure S2. Effect of *SlDCL1* silencing on *SlDCL2*-derived siRNAs accumulation.

Figure S3. Additional phenotypes of *FIL>>SlDCL1IR* plants.

Figure S4. Quantitative analysis of *SlDCL1* levels in *trans*-activated flowers.

Table S1. List of primers and probes used in this study.

Table S2. Summary of small RNA profiling.

Table S3. Summary of small RNA annotations.

Table S4. Summary of ShortStack-predicted miRNAs.

Table S5. List of ShortStack small RNA clusters.

Table S6. EdgeR results and differentially expressed miRNAs.

Supplementary Data
