# Supplementary material for: Global and local perturbation of the tomato microRNA pathway by a trans-activated DICER-LIKE 1 mutant
Source: J Exp Bot. 2013 Dec 27;65(2):725–39. doi: 10.1093/jxb/ert428 (PMC3904720; doi:10.1093/jxb/ert428)
Supplement: Supplementary Data [file supp_65_2_725__index.html]

Global and local perturbation of the tomato microRNA pathway by a trans-activated DICER-LIKE 1 mutant — Global and local perturbation of the tomato microRNA pathway by a trans-activated DICER-LIKE 1 mutant — Supplementary Data 

# Global and local perturbation of the tomato microRNA pathway by a *trans*-activated *DICER-LIKE 1* mutant

## Supplementary Data

Data files

**Files in this Data Supplement:**

- Supplementary Data - Supplementary Data
- Supplementary Data - Supplementary Data
- Supplementary Data - Supplementary Data
- Supplementary Data - Supplementary Data
