# Supplementary material for: Global and local perturbation of the tomato microRNA pathway by a trans-activated DICER-LIKE 1 mutant
Source: J Exp Bot. 2013 Dec 27;65(2):725–39. doi: 10.1093/jxb/ert428 (PMC3904720; doi:10.1093/jxb/ert428)
Supplement: Supplementary Data [file supp_ert428_jexbot110684_file001.pdf]

Journal of Experimental Botany, Global and local perturbation of the tomato microRNA pathway by a trans-activated DICER-LIKE 1 mutant

Michael Kravchik[1], Ramanjulu Sunkar [2], Subha Damodharan[1], Ran Stav[1], Matat Zohar[3], Tal Isaacson[3] and Tzahi Arazi[1,*]

Supplementary figures

Fig. S1 Sequence analysis of the tomato DCL1 homolog. (A) Scheme illustration of the gene, mRNA and protein structure of *SlDCL1*. In the gene, exons are represented by white boxes, introns and putative promoter and terminator sequences are shaded in black and dark grey, respectively. The location of the fragment used for silencing is indicated (*SlDCL1IR*). In the mRNA, 5' and 3' UTR sequences as determined by RACE are shaded in light grey and the predicted Watson-Crick pairing between sly-miR162 and target *SlDCL1* sequence is shown. Below, scheme of the predicted SlDCL1 protein structure compared to *Arabidopsis* (At) and rice (Os) DCL1s. The scheme shows in each protein the relative location of identified domains. (B) Accumulation of *SlDCL1* transcript in different tissues of tomato cv. Heinz based on published RNA-seq data from the tomato genome consortium 2012, Supplementary Table S1. Data are means of normalized expression of two independent biological samples. MG - mature green; Br - breaker.

Fig. S2 Effect of *SlDCL1* silencing on *SlDCL2*-derived siRNAs accumulation. Schematic of *35S>>SlDCL1IR* and control siRNAs accumulation at indicated *SlDCL2*. The normalized abundances of siRNAs were plotted relative to their mRNA sequence as a function of the positions of their 5' ends. Values above and below zero indicate siRNAs

matching the plus and minus strand, respectively. The miR6026 cleavage site is indicated. Bar = 21 nts.

Fig. S3 Additional phenotypes of *FIL>>SlDCL1IR* plants. From top to bottom, control (*FIL:LhG4*) and *FIL>>SlDCL1IR* 7 DAS seedlings, the width of the pot is 7 cm; Representative 30 DAS cotyledons; Representative 42 DAS plants, white arrowheads mark the position of representative axillary meristems, which are dormant and active in *FIL:LhG4* and *FIL>>SlDCL1IR* plants, respectively, asterisks mark leaves that were cut to enable better visualization of the nodes. Inset shows representative cotyledonary axillary meristem outgrowth at a higher magnification. Scale bar = 1 cm.

Fig. S4 Quantitative analysis of *SlDCL1* levels in transactivated flowers. QRT-PCR analysis of *SlDCL1* expression levels in (A) Less than 1 mm *AP1>>SlDCL1IR* buds (B) isolated *AP3>>SlDCL1IR* petals from 10 mm buds. TIP41 and CAC genes were used as reference genes in A and B, respectively. Error bars indicate ± SD of three biological replicates, each measured in triplicate. Asterisks indicate significant difference as determined by Student’s *t* test ($P \leq 0.01$).

A

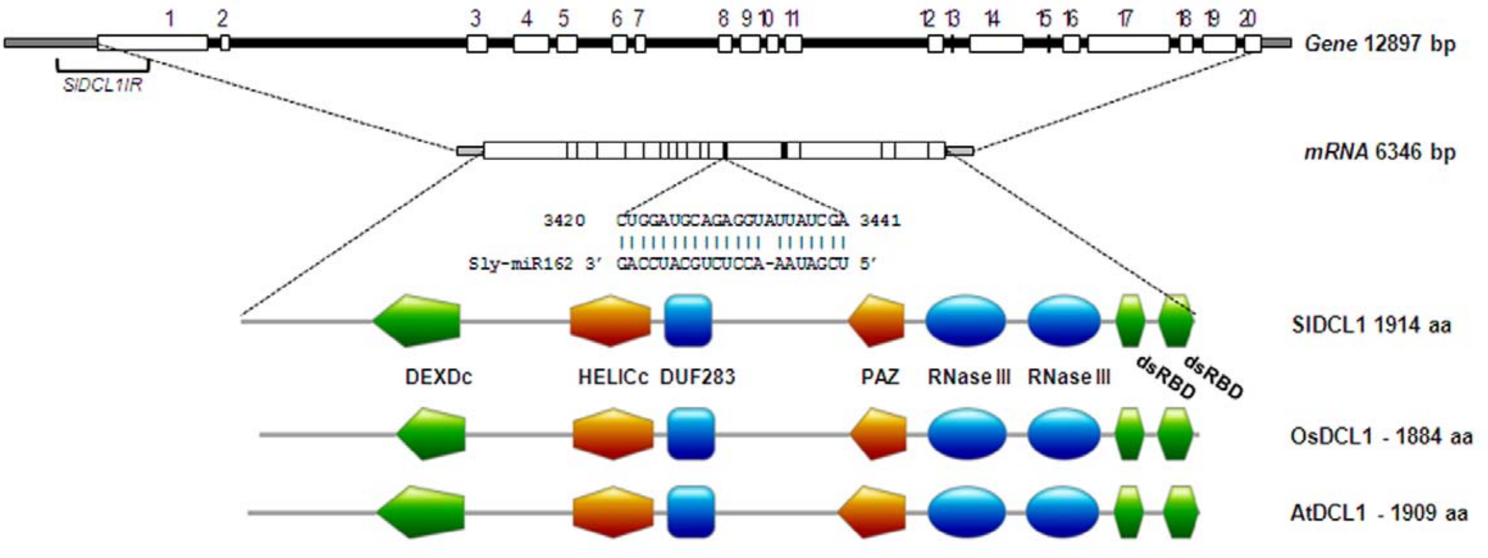
1
2
3
4
5
6
7
8
9
10
11
12
13
14
15
16
17
18
19
20
Gene 12897 bp
SlDCL1IR
mRNA 6346 bp
3420 CUGGAUGCAGAGGUAUUAUCGA 3441
Sly-miR162 3' GACCUACGUCUCCA-AAUAGCU 5'
SlDCL1 1914 aa
DEXDc
HELICc
DUF283
PAZ
RNase III
RNase III
dsRBD
dsRBD
OsDCL1 - 1884 aa
AtDCL1 - 1909 aa

B

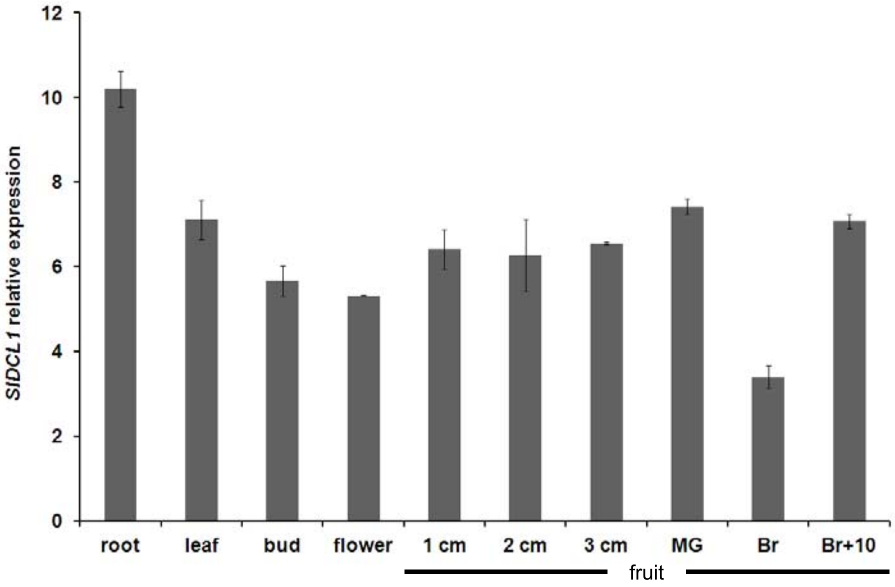
SlDCL1 relative expression
0
2
4
6
8
10
12
root
leaf
bud
flower
1 cm
2 cm
3 cm
MG
Br
Br+10
fruit

Figure S1

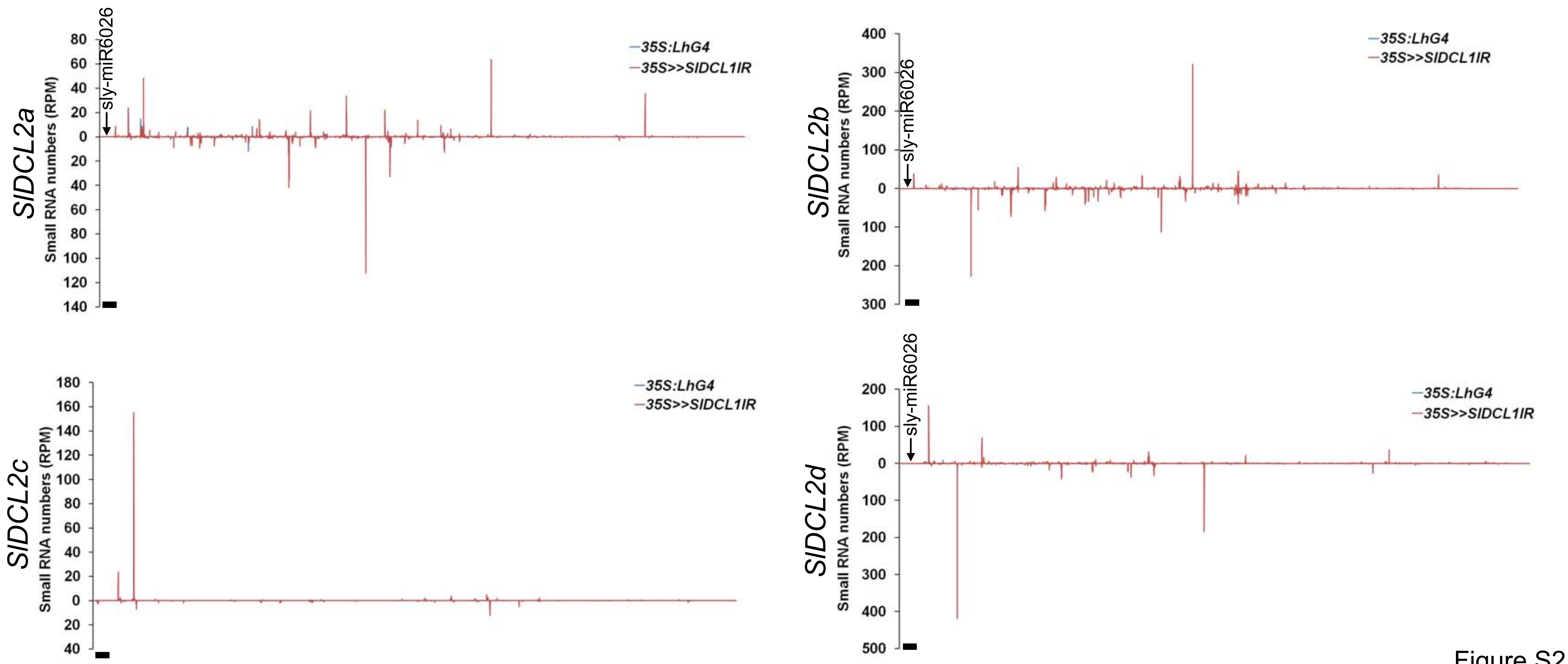

SlDCL2a
Small RNA numbers (RPM)
sly-miR6026
35S:LhG4
35S>>SlDCL1IR
80
60
40
20
0
20
40
60
80
100
120
140
SlDCL2b
Small RNA numbers (RPM)
sly-miR6026
35S:LhG4
35S>>SlDCL1IR
400
300
200
100
0
100
200
300
SlDCL2c
Small RNA numbers (RPM)
35S:LhG4
35S>>SlDCL1IR
180
160
140
120
100
80
60
40
20
0
20
40
SlDCL2d
Small RNA numbers (RPM)
sly-miR6026
35S:LhG4
35S>>SlDCL1IR
200
100
0
100
200
300
400
500

Figure S2

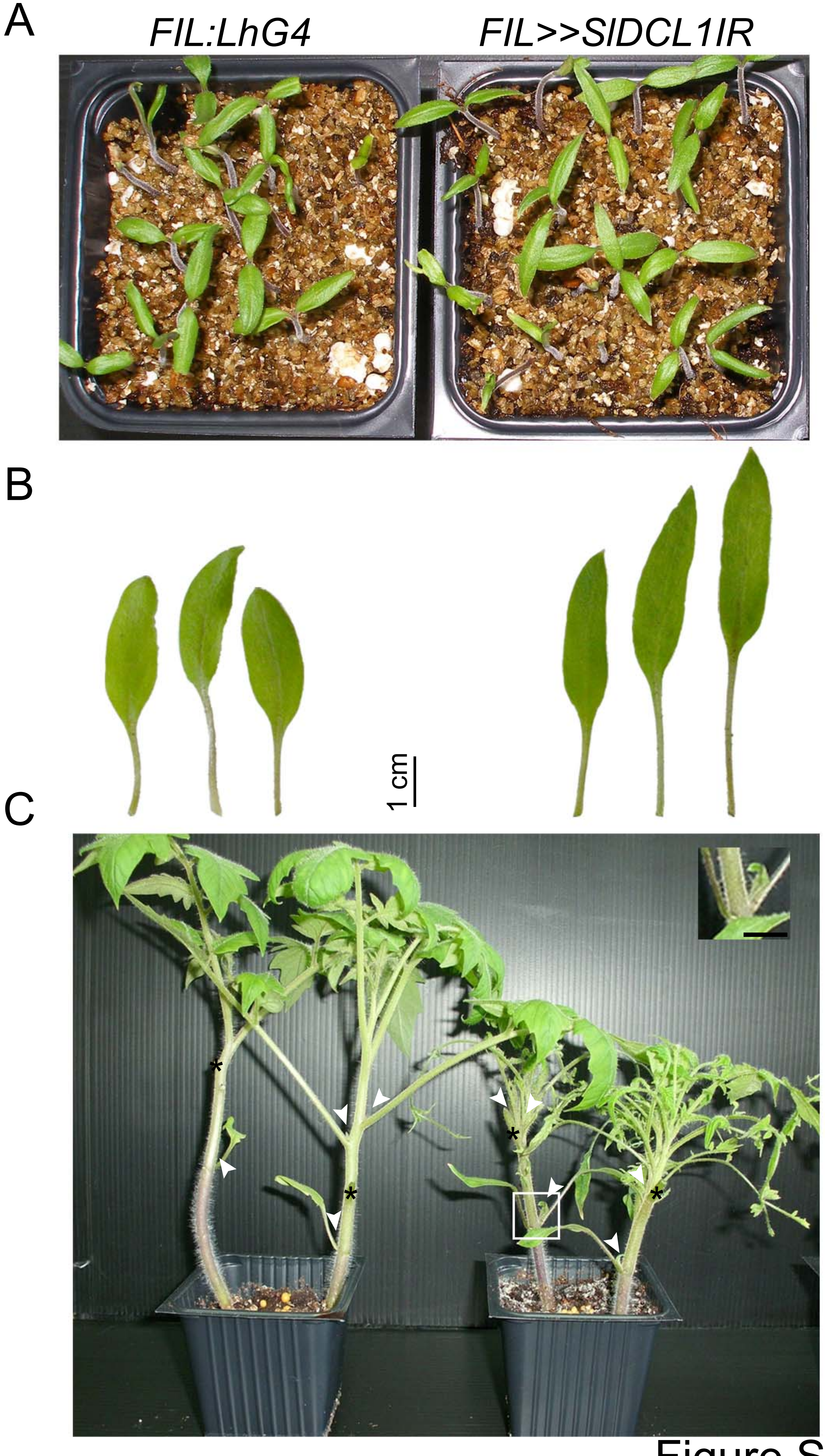

Figure S3

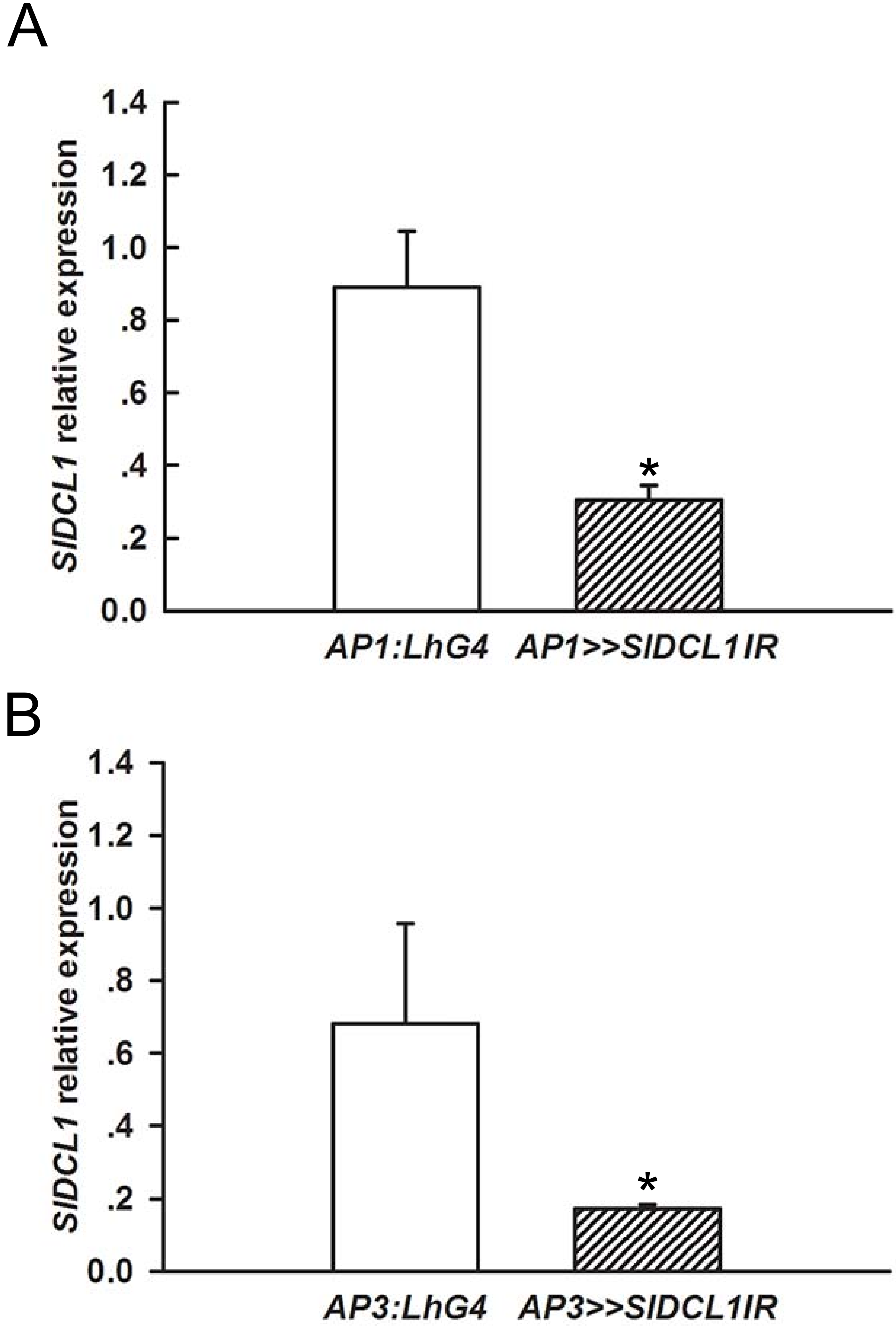
A
1.4
1.2
1.0
.8
.6
.4
.2
0.0
SlDCL1 relative expression
*
AP1:LhG4
AP1>>SlDCL1IR
B
1.4
1.2
1.0
.8
.6
.4
.2
0.0
SlDCL1 relative expression
*
AP3:LhG4
AP3>>SlDCL1IR

Figure S4

Supplementary Table S1. Primers and probes used in this study

**Primer ID**	**Primer sequence (5' – 3')**[abc]	**Remarks**
GeneRacer-5'	CGACTGGAGCACGAGGACACTGA	5' and 3' RACE
GeneRacer-5'-nested	GGACACTGACATGGACTGAAGGAGTA	
GeneRacer-3′	GCTGTCAACGATACGCTACGTAACG	
GeneRacer-3′-nested	CGCTACGTAACGGCATGACAGTG	
SlDCL1-5'-RACE	TTCAGCAGCGTCTGATAGAAAC	
SlDCL1-5'-RACE-nested	AAAGCCAAGGATTCCGCAGCAGCTCTTCTA	
SlDCL1-3'-RACE	TGTGGCCCGTTGTCCTTGCAAATTC	
SlDCL1-3'-RACE-nested	TCAAGAGTCTCATCCGGACCCAATTGAT	
SlDCL2a_F_28	CTGCTGGCAAACATCCTCTT	
SlDCL2d_R_280	GTCGGTATGCAACATCAGAGC	
SlDCL2b-5'-RACE	GCATCATCAGAGCATCTCCTTGCTGAGTCACTA	
SlDCL2b-5'-RACE-nested	TGCAAATGGCAAAGGATCAGCAGAGACTA	
SlDCL1IRRNA_R	CCCAAGCTTGAATTCCTCATAAGCATCAATCCAAT	SlDCL1 RNAi construct
SlDCL1IRDNA_F	ACGCGTCGACTGCAGACGATATTAAAGTCCGAAAGATCA	
SlDCL1IRRNA_F	GGTTGCTAGGTCTACAGGAGCA	
SlDCL1IRDNA_R	CAGCTTCAGTGGACTCTCTGGA	
SlDCL1_F_XhoI	CCGCTCGAGGGTTGCTAGGTCTACAGGAGCA	
SlDCL1_F_898	GTGGTCGTGGTGGAAGAGAT	SlDCL1 sequencing
SlDCL1_F_2481	AGTCAAGAAATGCGAACAGGTC	
SlDCL1_F_3227	GGATTTTGTCGGGAAAGGAT	
SlDCL1_F_4019	TGCAGCTCACTCTGGAAAGA	
SlDCL1_R_1892	CCTCTAGCTCCAGCATCACA	
SlDCL1_R_2685	TATAATCTGATCCGGGCTTACG	
SlDCL1_R_6168	TTCAGCAGCGTCTGATAGAAAC	
pFlap_intron_F	AATTTCTTGTTTCCGATCCTCATA	
OCS_rev	GAAACCGGCGGTAAGGATCT	Transgene identification
qRT-SGN-U592620_F	TTCAGGCCTCTGAACTATTGCT	*SlHAM*
qRT-SGN-U592620_R	CAACTGCAGAGCCTCCTTGATA	
qRT-Solyc03g115850_F	CCACCATTGACAGATTCATCG	
qRT-Solyc03g115850_R	GGTGAACGAAGTCGGAAGAG	NAC domain protein *(Solyc03g115850.2)*
qRT-SlARF3_F	AACTACATTTCTCCCTTCCAG	Real time primers
qRT-SlARF3_R	TCACAACAAACACCTGCTAC	
qRT-SlARF4_F	CGAAAGAACCATCTACTCC	Real time primers
qRT-SlARF4_R	AAAGCCTCTCCAACTCAAC	
qRT-SlARF10_F	CAGGTCCAGCAGTCCTTTCT	Real time primers
qRT-SlARF10_R	CGCTGGAAACTTGGTGGTAA	
qRT-TIP41_F	ATGGAGTTTTTGAGTCTTCTGC	Real time primers
qRT-TIP41_R	GCTGCGTTTCTGGCTTAGG	
qRT-SlDCL1_F	TCGAAGGACCCATTCTTAACTG	Real time primers
qRT-SlDCL1_R	CTATTGGCCCTCTGAAGACAAG	
qRT-SlDCL2a_F	CGGCTTCCGAAGAAGGTA	Real time primers
qRT-SlDCL2a_R	TTCAGCACTAGTCAAGAA	
qRT-SlDCL2b_F	TGCAGGACATATTTCTGTGGTC	Real time primers
qRT-SlDCL2b_R	TGAACATCCAAGCCCTCTTC	
qRT-SlDCL2d_F	GCTCAACTCTAAATTGGATTCCTG	Real time primers
qRT-SlDCL2d_R	AACTCTTTGAGTGTTCTTCAGTGTT	
qRT-SlDCL3_F	TTGCCACTGATGTGGTTGAG	Real time primers
qRT-SlDCL3_R	TCCCTCTGCTTCTTGTTTCC	
qRT-SlDCL4_F	AGTGAAGCTCAGATGGATGATG	Real time primers
qRT-SlDCL4_R	GCAGAAATACTCGTCGTGTGG	
qRT-SlCAC F	CCTCCGTTGTGATGATGTAACTGG	Real time primers
qRT-SlCAC R	ATTGGTGGAAAGTAACATCAT	
sly-miR160a_rc	TGGCATACAGGGAGCCAGGCA	sly-miR160a probe
sly-miR164_rc	TGCACGTGCCCTGCTTCTCCA	sly-miR164 probe
sly-miR171_rc	GATATTGGCACGGCTCAATCA	sly-miR171 probe
sRNA_id_1941824_rc	AGAGACACACTTATACTATACTAA	*TAPIR probe*
sly-miR390_rc	GGTGCTATCCCTCCTGAGCTT	sly-miR390 probe
sRNA_ID1897365_rc	AGTCTTTGCTCTAAGGCCACA	TAS5 probe
sRNA_ID477247_rc	GGCCCAAAGACAACATGAAGT	TAS4 probe

[a]Sequences corresponding to restriction enzyme sites are underlined.

Supplementary Table S2. Summary of small RNA profiling

	35S:LhG4-1	***35S:LhG4-2***	***35S>>SlDCL1IR-1***	***35S>>SlDCL1IR -2***
Raw Reads [a]	4,975,066	5,619,906	3,907,268	4,519,122
Genome-matched reads	3,487,457	4,008,933	2,730,156	3,223,011
Ratio [b]	70	71.3	70	71.3
t/r/sn/snoRNA	1,047,049	1,143,490	839,552	1,115,895
Ratio [c]	30.0	28.5	30.8	34.6
Available small RNA reads [d]	2,440,408	2,865,443	1,890,604	2,107,116
Unique sRNA sequences	899,019	1,139,600	854,896	923,666
Total hits on genome [e]	9,503,296	11,811,550	9,090,523	9,438,606

[a]Total acquired sequencing data from Illumina Hiseq 200 analyzer (18-26 nucleotide), 5' and 3' adaptors have been trimmed.

[b]The ratio represents the percentage of genome-matched reads to the raw reads.

[c] The ratio represents the percentage of t/r/sn/snoRNAs reads to the genome-matched reads.

[d]The reads excluding the t/r/sn/snoRNAs and unmapped reads.

[e] Sum of the hits of all the available unique small RNA sequence on related tomato genome.

Supplementary Table S3. Summary of small RNA annotations

	35S:LhG4-1	***35S:LhG4-2***	***35S>>SlDCL1IR-1***	***35S>>SlDCL1IR -2***
Available small RNA reads[a]	2,440,408	2,865,443	1,890,604	2,107,116
RNAi loci associated	175	17	6,601	7,136
Unannotated	1,574,705	1,864,450	1,363,737	1,466,600
cDNA associated	287,865	326,764	237,490	291,913
miRNA associated	252,851	313,790	72,018	101,023
Pre-miRNA associated	173,586	182,012	76,723	95,713
Repeat associated	151,226	178,410	134,035	144,731

[a] The reads excluding the t/r/sn/snoRNAs and unmapped reads (clean).
